# An Unusual Presentation of Henoch-Schönlein Purpura With Penile Involvement in a 10-Year-Old Boy From Mexico

**DOI:** 10.7759/cureus.90076

**Published:** 2025-08-14

**Authors:** Israel Silva Pizano, Jose Luis Maldonado Calderón, Rodrigo Romero Mata, Adrian Gutierrez Gonzalez, Cesia Gisela Avalos Fernandez, Jose Ricardo Guerra Rios, Rolando Lopez Rodriguez, Joel Ibarra De La Garza

**Affiliations:** 1 Urology, Hospital Universitario de Monterrey Dr. José Eleuterio González, Monterrey, MEX; 2 Rheumatology, Hospital Universitario de Monterrey Dr. José Eleuterio González, Monterrey, MEX

**Keywords:** first case, henoch-schönlein purpura (iga vasculitis), iga vasculitis, penile involvement, vasculitis in children

## Abstract

Immunoglobulin A (IgA) vasculitis, also known as Henoch-Schönlein purpura, is the most common systemic vasculitis in childhood. It primarily affects the skin, joints, gastrointestinal tract, and kidneys. Extrarenal genitourinary manifestations are uncommon, and penile involvement is exceptionally rare, with very few cases documented worldwide and, to our knowledge, none previously reported in Mexico.

A previously healthy 10-year-old boy presented with a purpuric rash on the extremities, arthralgia, abdominal pain, and progressive penile edema. Physical examination revealed pharyngeal hyperemia, palpable purpuric lesions on the extremities, an edematous and tender penis, and scrotal papules. Laboratory studies were unremarkable, with no evidence of infectious or autoimmune processes. Testicular ultrasound excluded torsion or other surgical causes. A clinical diagnosis of IgA vasculitis with penile involvement was made, and treatment was initiated with intravenous methylprednisolone followed by oral prednisone, achieving complete resolution without sequelae.

Genital involvement in Henoch-Schönlein purpura is rare and may mimic surgical urological conditions. Early recognition of this presentation helps avoid unnecessary interventions. Doppler ultrasound and clinical correlation are essential for diagnosis. In the absence of severity criteria, corticosteroid therapy combined with local supportive measures is usually effective.

This case represents the first report in Mexico of Henoch-Schönlein purpura with penile involvement. It highlights the importance of considering this entity in the differential diagnosis of pediatric acute scrotum and demonstrates the effectiveness of conservative management.

## Introduction

Immunoglobulin A (IgA) vasculitis, also known as Henoch-Schönlein purpura, is a systemic vasculitis affecting small-caliber blood vessels, primarily involving the skin, joints, gastrointestinal tract, and kidneys [1-3]. Less frequently, it may present with manifestations in the central nervous system, lungs, and scrotum [2]. These atypical localizations have been reported less commonly and may pose diagnostic and therapeutic challenges.

This disease mainly affects the pediatric population, with an estimated incidence of 10 to 20 cases per 100,000 children per year, being more common in boys under 10 years of age [3,4]. In adults, the clinical presentation tends to be more severe, with a higher risk of progression to chronic kidney disease.

The exact etiology of IgA vasculitis is not fully understood, although an interaction between environmental, genetic, and antigenic factors has been postulated [3]. In more than 30% of cases, a preceding infection by group A *Streptococcus*, particularly of the upper respiratory tract, has been identified as an immunological trigger [2].

Pathophysiologically, it is suggested that vasculitis is caused by the formation of IgA-antigen immune complexes in response to infectious or pharmacologic stimuli. These complexes deposit in the walls of small blood vessels, triggering an inflammatory cascade mediated by prostaglandins, cytokines, and activation of the complement system via the C3 receptor on lymphocytes [3,5].

Although most cases of IgA vasculitis are considered self-limiting, the severity of symptoms can vary. Penile involvement is an unusual clinical manifestation, with few documented reports, which limits the understanding of its progression, treatment, and prognosis. In this context, we present the case of a pediatric patient with IgA vasculitis and penile involvement, with the aim of contributing to the existing literature and enhancing the understanding of this atypical presentation. Informed consent for the publication of this case was obtained from the parents.

## Case presentation

A 10-year-old male patient, previously healthy, with an unverified but reportedly complete vaccination schedule and appropriate neurodevelopment, presented with an initial clinical picture of abdominal pain and two episodes of vomiting seven days prior to admission, which resolved after paracetamol administration. Three days before admission, he developed pharyngodynia, followed by an erythematous maculopapular rash on the extremities and genital region, accompanied by unquantified fever. The next day, palpable purpuric lesions appeared on the forearms and hands, along with arthralgia affecting the elbows, wrists, and phalanges. Subsequently, the purpuric lesions extended to the lower limbs. He then developed progressive penile edema, which prompted hospital evaluation.

**Figure 1:**
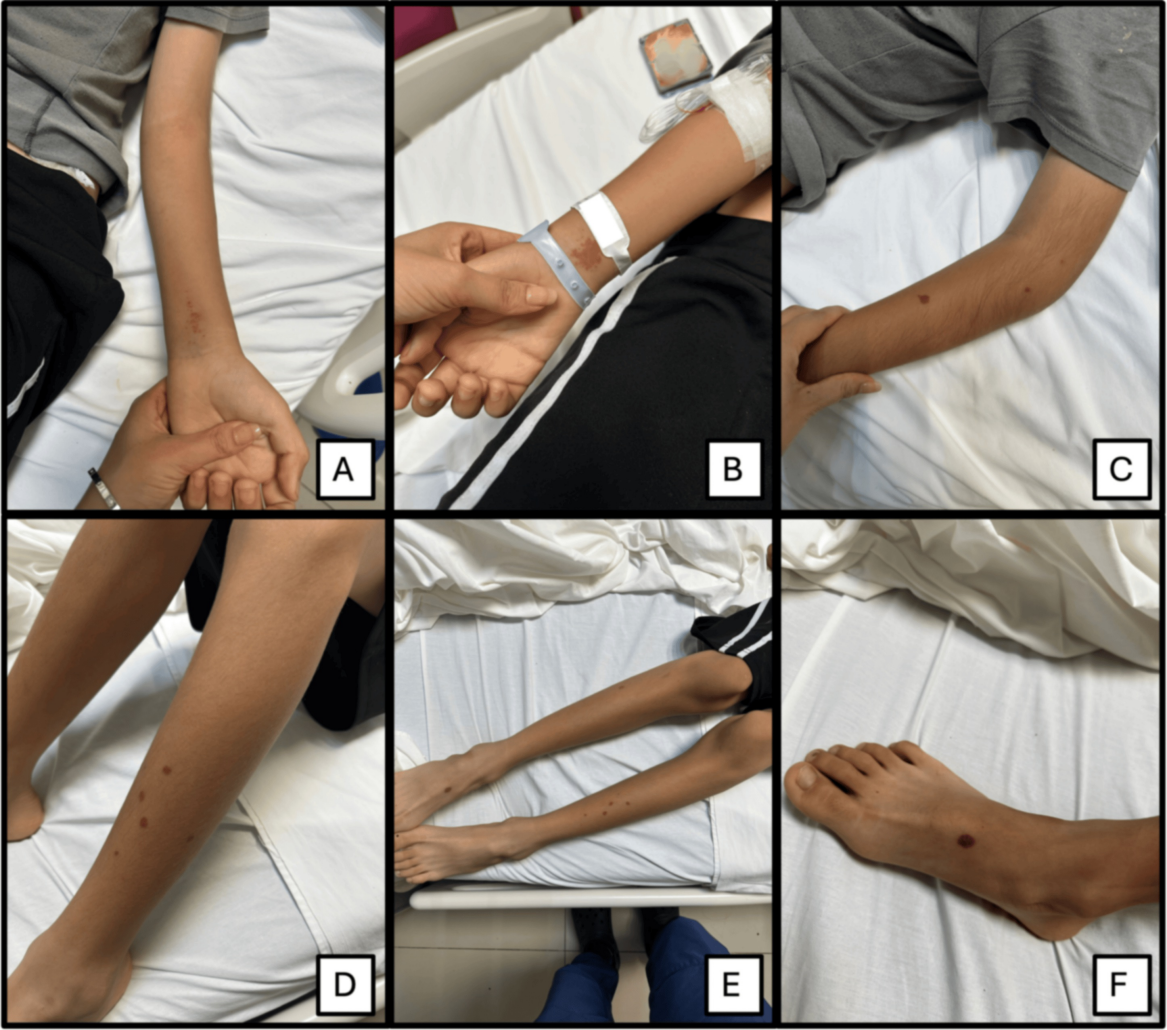
Purpuric cutaneous lesions in a pediatric patient diagnosed with IgA vasculitis (Henoch-Schönlein purpura). A. Scattered linear purpuric lesions on the volar aspect of the left forearm. B. Purpuric erythema on the dorsal aspect of the right wrist. C. Isolated purpuric papules on the extensor aspect of the left forearm. D. Multiple linear and scattered purpuric macules on the left leg. E. Bilateral distribution of the lesions on the lower extremities, predominantly on the anterior aspect. F. Well-demarcated purpuric macule on the dorsum of the right foot.

On admission, the patient was hemodynamically stable, well hydrated, and with normal vital signs. Physical examination revealed pharyngeal hyperemia with tonsillar exudate, and disseminated dermatosis on the extremities and scrotal region (Figure 1).

**Figure 2:**
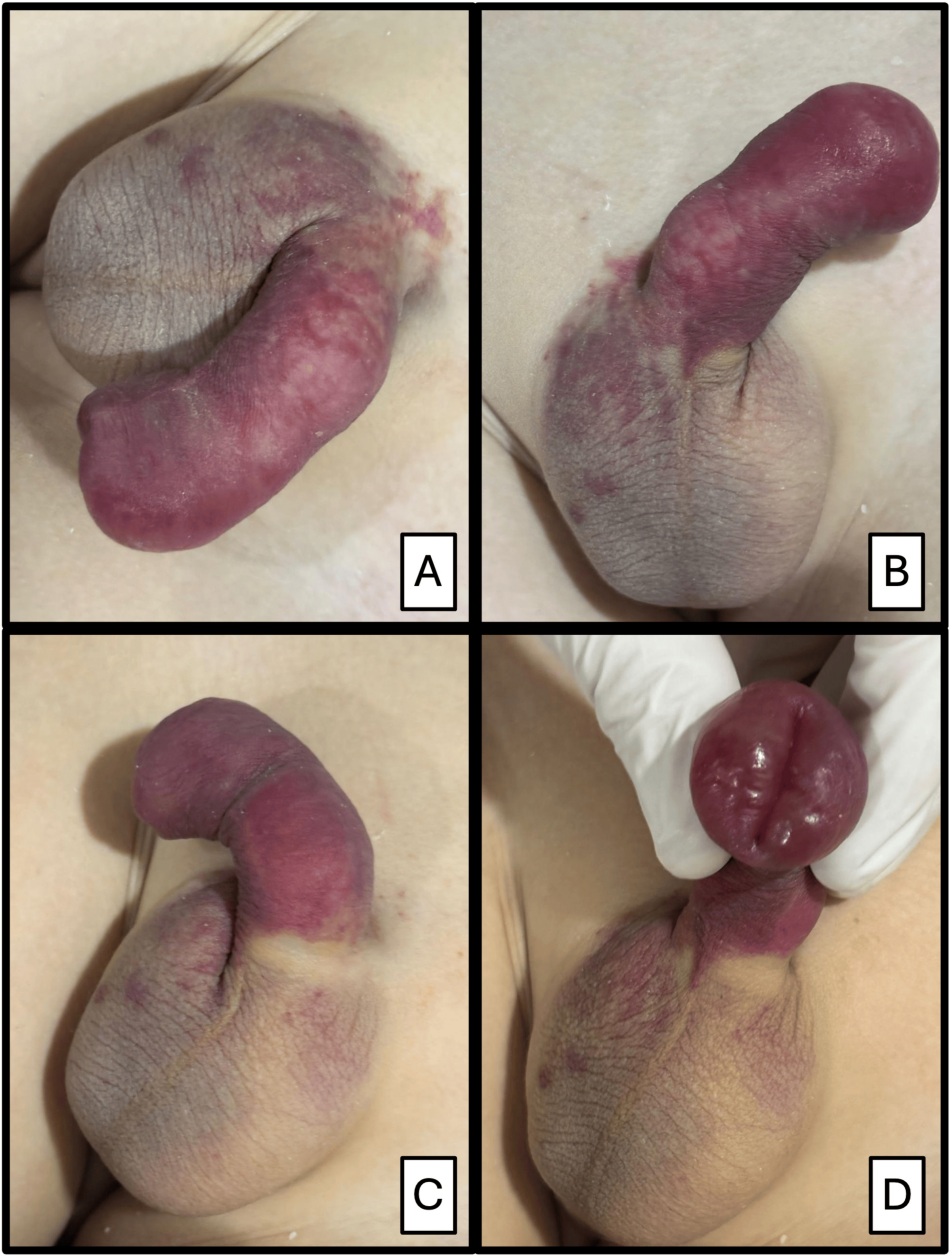
Penile involvement in a pediatric patient with IgA vasculitis (Henoch-Schönlein purpura). A. Right lateral view of the penis showing diffuse edema, intense erythema, and purpuric lesions on the foreskin and penile base, extending into the right scrotum. B. Left lateral view of the penis showing uniform thickening of the foreskin and penile shaft, with violaceous erythema throughout. C. Inferior oblique view showing penile and scrotal edema with no evidence of fluctuation or discharge. D. Frontal view with retraction of the foreskin, showing marked congestion of the foreskin and purpuric erythema.

Abdominal examination showed localized tenderness in the right flank upon deep palpation. External genital examination revealed a male phenotype with Tanner stage I, uncircumcised penis with retractable foreskin, diffuse erythema and edema involving all penile segments, local hyperthermia, and tenderness on palpation without discharge. The scrotum showed mild swelling with erythematous papules; testes were in the scrotal sac without palpable abnormalities (Figure 2). 

Laboratory studies revealed hemoglobin 17.1 g/dL, leukocytes 7.47 × 10³/mm³, platelets 368 × 10³/mm³, with renal function, electrolytes, and liver function tests within normal ranges. Urinalysis showed turbid urine, pH 8.0, specific gravity 1.018, without relevant infectious findings. Given the unusual presentation, autoimmune serologies were requested, all of which were negative (ANA, anti-dsDNA, ANCA, among others), with normal complement levels (C3: 179 mg/dL, C4: 40 mg/dL) (Table 1). 

**Table 1:** Laboratory findings on admission. All values were within or near normal limits. Autoimmune panel was negative, supporting the diagnosis of IgA vasculitis without systemic autoimmune disease. Complement levels were within normal range. BUN: blood urea nitrogen; AST: aspartate aminotransferase; ALT: alanine aminotransferase; ALP: alkaline phosphatase; LDH: lactate dehydrogenase; ANA: antinuclear antibody; anti-dsDNA: anti–double-stranded DNA antibody; ANCA: antineutrophil cytoplasmic antibody; anti-SSA/Ro: anti–Sjogren’s syndrome-related antigen A/Ro antibody; anti-SSB/La: anti–Sjogren’s syndrome-related antigen B/La antibody; anti-Sm: anti-Smith antibody; anti-RNP: anti–ribonucleoprotein antibody; C3: complement component 3; C4: complement component 4

Test	Result	Normal values
Hemoglobin	17.1 g/dL	12.2 – 18.1 g/dL
Leukocytes	7.47 x10^3/µL	4.0 – 11.0 x10^3/µL
Platelets	368 x10^3/µL	142 – 424 x10^3/µL
BUN	20 mg/dL	7 – 20 mg/dL
Creatinine	0.4 mg/dL	0.6 – 1.4 mg/dL
Serum chloride	98.5 mmol/L	101 – 110 mmol/L
Serum sodium	135.5 mmol/L	135.0 – 145.0 mmol/L
Serum potassium	5.0 mmol/L	3.6 – 5.0 mmol/L
AST	19 U/L	10 – 42 U/L
ALT	8 U/L	10 – 42 U/L
ALP	138 U/L	38 – 126 U/L
LDH	137 U/L	91 – 180 U/L
ANA	NEGATIVE	NEGATIVE
ANTI-dsDNA	<10 U/mL	<100 U/mL
ANCA	<2 U/mL	<20 U/mL
ANTI-SSA/Ro	<2 U/mL	<20 U/mL
ANTI-SSB/La	<2 U/mL	<20 U/mL
ANTI-Sm	<2 U/mL	<20 U/mL
ANTI-RNP	<2 U/mL	<20 U/mL
C3	179 mg/dL	90 – 180 mg/dL
C4	40 mg/dL	10 – 40 mg/dL

**Figure 3:**
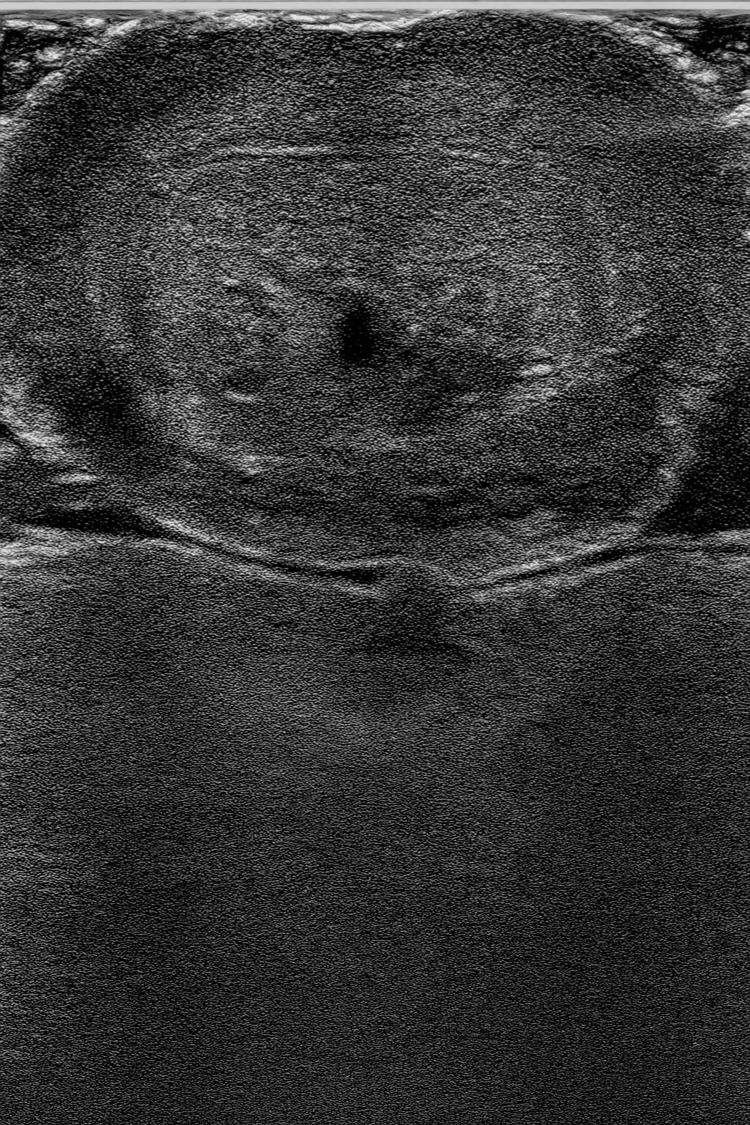
Axial section of the penis ultrasound showing edema of adjacent cellular tissue.

Scrotal and penile ultrasound showed both testes within the inguinal canal with preserved peripheral blood flow, without evidence of torsion, cellulitis, or fluid collections. The penis exhibited edema without structural abnormalities and a patent urethra. Abdominal ultrasound revealed no pathological findings (Figure 3).

After pediatric rheumatology evaluation, a diagnosis of IgA vasculitis with atypical genital involvement was established. Management was initiated with naproxen and intravenous methylprednisolone (2 mg/kg/day) followed by an oral prednisone tapering regimen (50 mg/day), in addition to physical measures including intermittent local ice application and maintaining the glans at a 90° angle.

The patient showed adequate clinical resolution, with a total hospital stay of three days and follow-up through outpatient consultation. 

## Discussion

IgA vasculitis (Henoch-Schönlein purpura) is the most common systemic vasculitis in pediatrics, with 90% of patients presenting before the age of 10 years (mean age of onset of six years) and a male-to-female ratio of 2:1 [4,6,7]. Population-based studies have demonstrated that Henoch-Schönlein purpura varies by geographic region and exhibits seasonal peaks, possibly due to its association with infectious diseases. In the Northern Hemisphere, the incidence of IgA vasculitis predominates between November and January [4]. Although it most commonly manifests as a single episode without long-term consequences, patients may develop renal involvement, which rarely progresses to end-stage renal disease or even death [5,8].

Extrarenal genitourinary involvement is uncommon, affecting 10-20% of patients with Henoch-Schönlein purpura; however, genital involvement, particularly penile inflammation, is extremely rare and scarcely documented [9-11]. While scrotal edema can occur in up to 20% of cases, penile inflammation has only been reported in a very limited number of pediatric patients worldwide [12]. Furthermore, to the best of our knowledge, there have been no reports in Mexico of IgA vasculitis with penile involvement.

Up to 50% of children present gastrointestinal symptoms ranging from mild (abdominal pain, nausea, vomiting) to severe complications such as gastrointestinal bleeding, obstruction, or perforation due to small-vessel vasculitis. IgA nephropathy has been reported in 20-54% of children with IgA vasculitis and is more common in older children [4]. Despite classic clinical manifestations such as abdominal pain, palpable purpura, and arthralgias, penile involvement as described in our case may lead to diagnostic confusion, mimicking local infections, orchiepididymitis, urethritis, trauma, sexual abuse, or even urological surgical emergencies such as testicular torsion [4,12-14]. In addition to maintaining a high index of suspicion in such presentations, a thorough physical examination, recognition of cutaneous purpura, and Doppler scrotal ultrasound are key to avoiding unnecessary interventions, as up to 3% of pediatric patients presenting with acute scrotum in emergency settings may have IgA vasculitis [15].

There is no single diagnostic test for IgA vasculitis; diagnosis is based on clinical criteria and laboratory findings. The current diagnostic criteria for IgA vasculitis are those established by EULAR/PRINTO/PRES, which state that in a pediatric patient with purpura (round, oval, or retiform) predominantly on the lower extremities, the diagnosis is confirmed if at least one of the following four criteria is present: (1) abdominal pain, (2) histological evidence of IgA deposition, (3) arthritis or arthralgia, or (4) renal involvement. This classification offers 100% sensitivity and 87% specificity for IgA vasculitis [5,16]. Although 50-70% of patients may have elevated serum IgA levels, this finding lacks diagnostic specificity. Immunological tests, such as ANA and rheumatoid factor, are typically negative, and complement levels, including C3 and C4, remain within normal limits [4,16]. Skin biopsy is not necessary for typical lesions predominantly affecting the lower extremities and buttocks [16]. In cases of severe abdominal pain, an abdominal ultrasound can be performed to rule out intussusception, a complication of this disease [16]. Kaya Akca U et al. reported two cases of IgA vasculitis with penile involvement, in which the diagnosis was made solely on clinical manifestations and the disease course [17]. In our case, the diagnosis was made clinically, as characteristic lesions and normal laboratory findings supported the diagnosis, and a skin biopsy was not required. Despite the absence of severe abdominal pain, an abdominal ultrasound was performed to exclude associated complications such as intussusception.

Treatment is mainly aimed at symptom control, with appropriate analgesia for arthropathy or abdominal pain, as well as adequate hydration if nonsteroidal anti-inflammatory drugs are required [16]. In the absence of severe gastrointestinal or renal complications, the disease is usually self-limiting, resolving within approximately 14 days [18,19]. Although evidence supporting its use is limited, corticosteroids are indicated in cases of extensive skin lesions, orchitis, cerebral vasculitis, pulmonary hemorrhage, or severe organ damage, with a recommended dose of 1-2 mg/kg/day of prednisone [16]. There are no specific guidelines for the urological management of penile involvement in IgA vasculitis. In their report, Sandell et al. managed a case with high-dose pulse methylprednisolone followed by a 96-hour course of oral prednisolone, resulting in a rapid response and immediate improvement [10]. In our case, treatment with methylprednisolone and oral prednisone, in addition to measures such as intermittent local ice application and maintaining the penis at a 90° angle, led to rapid improvement without sequelae.

The prognosis of IgA vasculitis is generally favorable. Approximately one-third of cases resolve spontaneously in less than 14 days; however, recurrence has been described in up to one-third of patients within four months after symptom resolution [20]. Annual follow-up is recommended in patients with persistent microscopic hematuria due to the risk of long-term renal deterioration. However, in cases such as ours, where urinalysis results are normal, further follow-up is not considered necessary [21].

## Conclusions

This case represents, to the best of our knowledge, the first documented report in Mexico of Henoch-Schönlein purpura with penile involvement. Its unusual presentation highlights the importance of considering this entity in the differential diagnosis of acute pediatric urological conditions, such as acute scrotum or penile inflammation of infectious origin. Timely clinical diagnosis, complemented by imaging studies and the appropriate use of corticosteroids, allowed for rapid resolution without sequelae. Although genital involvement may raise concern due to its apparent severity, the prognosis is generally favorable when early conservative management is implemented, with a low rate of complications or recurrence in the absence of significant renal involvement.
